# Inhibitory effect of Samul-tang on retinal neovascularization in oxygen-induced retinopathy

**DOI:** 10.1186/s12906-015-0800-7

**Published:** 2015-08-12

**Authors:** Yun Mi Lee, Chan-Sik Kim, Kyuhyung Jo, Eun Jin Sohn, Jin Sook Kim, Junghyun Kim

**Affiliations:** Korean Medicine-Based Herbal Drug Development Group, Herbal Medicine Research Division, Korea Institute of Oriental Medicine (KIOM), 1672 Yuseongdaero, Yuseong-gu, Daejeon, 305-811 South Korea

**Keywords:** Retinal neovascularization, Stromal cell-derived factor 1, Chemokine (C-X-C motif) receptor 4, Vascular endothelial growth factor, Oxygen-induced retinopathy

## Abstract

**Background:**

Retinal neovascularization is a common cause of vision loss in proliferative diabetic retinopathy, retinopathy of prematurity and age-related macular degeneration. Samul-tang (SMT) is a widely used traditional herbal medicine in East Asia and is also known as Shimotsu-to in Japanese and Si-Wu decoction in Chinese. This study was designed to evaluate the inhibitory effect of SMT on retinal pathogenic angiogenesis in a mouse model of oxygen-induced retinopathy (OIR).

**Method:**

The mice were exposed to a 75 % concentration of oxygen for five days, starting on postnatal day 7 (P7-P12). The mice were then exposed to room air and were intraperitoneally injected with SMT (10 mg/kg or 50 mg/kg) once per day for five days (P12-P16). On P17, we measured retinal neovascularization and evaluated both the expression of angiogenesis-related proteins and changes in the gene expression level in the mRNA.

**Results:**

SMT reduced the area of the central retina and reduced retinal neovascularization in OIR mice. The protein array revealed that SMT reduced the level of SDF-1 protein expression. Quantitative real-time PCR revealed that the HIF-1α, SDF-1, CXCR4 and VEGF mRNA levels in the retinas of OIR mice were elevated compared with those of normal control mice. However, SMT decreased the levels of HIF-1α, SDF-1, CXCR4 and VEGF mRNA in OIR mice.

**Conclusion:**

We are the first to elucidate that SMT inhibits the retinal pathogenic angiogenesis induced by ischemic retinopathy in OIR mice. SMT significantly inhibited retinal neovascularization by downregulating HIF-1α, SDF-1, CXCR4 and VEGF. Based on the results of our study, SMT could be a useful herbal medicine for treating ischemic retinopathy.

## Background

Retinal neovascularization, which is the pathological growth of new blood vessels, is a characteristic feature of several eye diseases that cause catastrophic loss of vision. Proliferative diabetic retinopathy, retinopathy of prematurity and age-related macular degeneration are the most common causes of retinal neovascularization [1, 2].

Vascular endothelial growth factor (VEGF) plays a central pathogenic role in retinal neovascularization caused by hypoxia-induced retinal damage. Hypoxia in the retina induces compensatory changes in blood flow, the overexpression of cytokines and angiogenesis [3, 4]. The activation of VEGF promotes the migration and proliferation of endothelial cells with the formation of new blood vessels [5].

The disruption of VEGF signaling is a useful target for therapeutic interventions, and VEGF antagonists have an inhibitory effect on angiogenesis [6]. All anti-VEGF therapies require repeated injections of expensive VEGF antagonists and only offer a temporary respite from vascular leakage; thus, such therapies can achieve only partial clinical success. The lack of efficacy of anti-VEGF treatments may be caused by the effects of this treatment on the HIF pathway-mediated expression of other pro-angiogenic factors, such as stromal cell-derived factor 1 (SDF-1), platelet-derived growth factor-B (PDGF), insulin-like growth factor 1 (IGF-1), erythropoietin, and other factors [7, 8].

Stromal cell-derived factor (SDF)-1 is a member of the CXC subfamily of chemokines; it was initially cloned from murine bone marrow and was characterized as a pre-B cell growth stimulating factor [9]. Two isoforms of this factor (i.e., SDF-1α/CXCL12a and SDF-1β/CXCL12b) have been identified. These isoforms are encoded by a single gene and arise via alternative splicing [10]. CXCR4, which is a 7-transmembrane spanning G protein-coupled receptor and a known receptor for SDF-1, induces extravasation, locomotion, invasion, migration, homing, and cell survival [11, 12]. CXCR4 is selectively expressed in vascular endothelial cells and is upregulated by the angiogenic factors basic fibroblast growth factor (bFGF) and VEGF [13]. SDF-1, which is one of the most important chemokines induced by ischemia, and CXCR4 regulate specific steps in new vessel formation, proliferation and angiogenesis [14, 15].

Samul-tang (SMT), which is known as Shimotsu-to in Japanese and Si-Wu decoction in Chinese, is a basic prescription that consists of four herbs. For hundreds of years in Eastern Asia, SMT has been a traditional treatment for relieving pain, promoting blood circulation, and improving blood deficiencies, gynecological diseases and chronic inflammation [16, 17]. The novel pharmacological activities of SMT, including anti-cancer properties, anti-inflammatory effects and anti-pruritic activity, have recently been reported [17]. SMT has also been used because of its angiogenesis [18] and anti-diabetic properties [19]. Although retinal neovascularization is recognized as the hallmark of proliferative diabetic retinopathy, the effect of SMT on retinal pathogenic angiogenesis has not been reported. Therefore, the purpose of this study was to investigate the effect of SMT on retinal neovascularization in a mouse model of oxygen-induced retinopathy (OIR).

## Methods

### Preparation of SMT

A standardized SMT powder was kindly provided by Dr. Hyeun-Kyoo Shin (Korea Institute of Oriental Medicine, Daejeon, Korea). The formula for SMT consists of a mixture of four herbs, including *Angelica gigas* (4.6875 g), *Cnidium officinale* (4.6875 g), *Paeonia lactiflora* (4.6875 g), and *Rehmannia glutinosa* (4.6875 g). SMT was boiled with distilled water at 100 °C for 2 h, and the extract was condensed by freeze-drying (yield: 33.3 %). In a previous report, HPLC analysis identified that the major compounds of SMT are 5-hydroxymethyl-2-furaldehyde (5-HMF), albiflorin, paeoniflorin, ferulic acid, and nodakenin [17].

### A mouse model of oxygen-induced retinopathy

Ischemic retinopathy was induced in C57BL/6 mouse pups, as described previously [20]. On postnatal day 12 (P12), after being exposed to 75 ± 2 % oxygen for five days (P7-P12), the mice were randomly assigned to one of three groups: OIR group, SMT-10 (10 mg/kg/day) and SMT-50 (50 mg/kg/day). The normal control group (Con) was maintained under room air from birth until postnatal day 17 (P0 to P17). To minimize the differences in the pups’ weights, one mouse nursed 6–8 pups, and low-weight pups were discarded from the data sets. In humans, the recommended daily dose of SMT is approximately 18.75 g of dried herbs [21], which is equivalent to 6.24 g of the SMT extract (yield = 33.3 %). Considering a mean body weight of 60 kg for an adult, this dose for humans is equivalent to 104 mg/kg of SMT extract. We chose two doses of SMT (10 and 50 mg/kg) based on the minimum human equivalent dosage of raw herbs. The SMT was dissolved in saline, and 100 μl of this solution was injected intraperitoneally once per day for five days (P12-P16). The OIR and normal control groups were injected with the saline solution for five days. On P17, after five days of intraperitoneal injections, the mice were anesthetized and sacrificed. These experiments were repeated four times using four animals in each group. All experiments that used animals were approved by the Korea Institute of Oriental Medicine Institutional Animal Care and Use Committee.

### Fluorescein-dextran microscopy

On P17, the mice were deeply anesthetized using zolazepam (Zoletil, Virbac, Carros, France). PBS containing fluorescein-dextran (FD40S, Sigma, MO, USA) was subsequently circulated through the left ventricle. The retinas were dissected, flat mounted onto glass slides and viewed using fluorescence microscopy (BX51, Olympus, Tokyo, Japan). Quantification of the non-perfusion area was performed as described previously [22]. Briefly, images of the retina were taken at 40× magnification and imported into Adobe Photoshop. The area of non-perfusion in the center of the retina was quantified by comparing the number of pixels in the affected areas with the total number of pixels using the ImageJ software (NIH, MD, USA).

### Lectin staining

The retinas were incubated with 1 % bovine serum albumin and 5 % Triton X-100 in PBS for 3 h at room temperature. The retinas were washed 3 times with PBS and incubated overnight at 4 °C with biotin-conjugated isolectin B4 from *Bandeiraea simplicifolia* (Sigma-Aldrich, MO, USA) diluted 1:50 in PBS. The retinas were washed with 0.05 % Tween 20 in PBS, followed by incubation with streptavidin TRITC (1:500, Serotec, Oxford, UK) for 4 h at 37 °C. The retina was flat mounted and viewed using fluorescence microscopy (BX51, Olympus, Tokyo, Japan). The neovascular tufts in the retina were measured in the same way as the non-perfusion area.

### Angiogenesis-related protein array

To analyze the angiogenesis-related protein profiles in the retinas, a mouse angiogenesis array (R&D Systems, MN, USA) was performed following the manufacturer’s protocol. Briefly, the pooled mice retinas (*n* = 3) were homogenized in PBS using protease inhibitors and centrifuged at 10,000 × g for 5 min, and the total protein concentrations were quantified. The lysates were added to a membrane spotted with antibodies against angiogenesis-related proteins. After being incubated overnight at 4 °C, the membranes were treated with streptavidin-horseradish peroxidase and visualized using an enhanced chemiluminescence detection system (Amersham Bioscience, NJ, USA) on an image analyzer (LAS-3000, Fujifilm, Tokyo, Japan). Optical density measurements were obtained using the ImageJ software (NIH, MD, USA).

### Real-time PCR analysis

Total RNA was isolated using the TRIzol reagent (Invitrogen, CA, USA), and 0.5 μg of total RNA was reverse transcribed into cDNA with the PrimeScript First Strand cDNA Synthesis kit (Bio-Rad, CA, USA). Real-time quantitative PCR was performed with specific primers for HIF-1α, SDF-1, CXCR4, VEGF and GAPDH using an iQ5 Continuous Fluorescence Detector System (Bio-Rad, CA, USA). The sequences of the primers were as follows: for HIF-1α, 5′-GTCGGACAGCCTCACCAAACAG-3′ and 5′-TAGGTAGTGAGCCACCAGTCATCCAAGGAA-3′; for VEGF, 5′-TCCTCCTATCTCCACCACCTATCC-3′ and 5′-GACCCAGCCAGCCATACCC-3′; for SDF-1, 5′-GTCCACCTCGGTGTCCTCTT-3′ and 5′-GGGCACAGTTTGGAGTGTTG-3′; for CXCR4, 5′-TACCTCGCTATTGTCCACGC-3′ and 5′-GTGCACGATGCTCTCGAAGT-3′; for GAPDH, 5′-AACGACCCCTTCATTGAC-3′ and 5′-TCCACGACATACTCAGCAC-3′. All real-time PCR experiments were run in triplicate. The mRNA levels of GAPDH were determined for the normalization of the HIF-1α, SDF-1, CXCR4 and VEGF mRNA expression values using the iQ5 optical system software (Bio-Rad, CA, USA).

### Statistical analysis

The results were expressed as the mean ± SE and analyzed using one-way analysis of variance (ANOVA) followed by Tukey’s multiple comparison tests or using unpaired Student’s *t-*test. All analyses were performed using the Prism 6.0 software (GraphPad Software, San Diego, CA, USA).

## Results

### Effect on retinal neovascularization

**Fig. 1:**
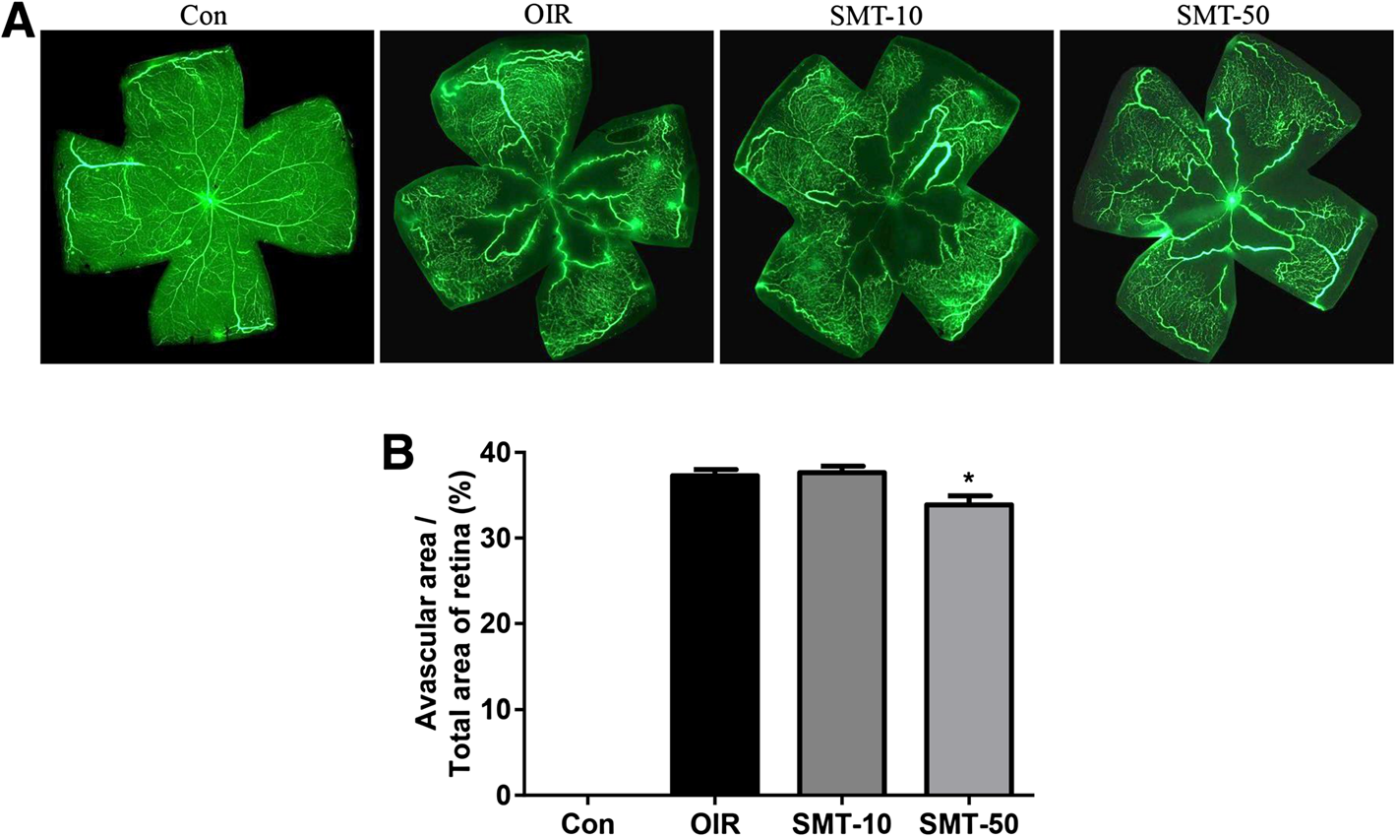
The effect of SMT on retinal neovascularization in OIR mice. **a** The retinal blood vessels were visualized via fluorescein angiography using FITC-dextran. Con, normal control mice; OIR, saline-treated OIR mice; SMT-10, OIR mice treated with 10 mg/kg of SMT; and SMT-50, OIR mice treated with 50 mg/kg of SMT. **b** The quantification results are expressed as the percentage of the central non-perfused area within the total retinal area. The values in the bar graph represent the mean ± SE (*n* = 5; *p < 0.05 vs. saline-treated OIR mice)

**Fig. 2:**
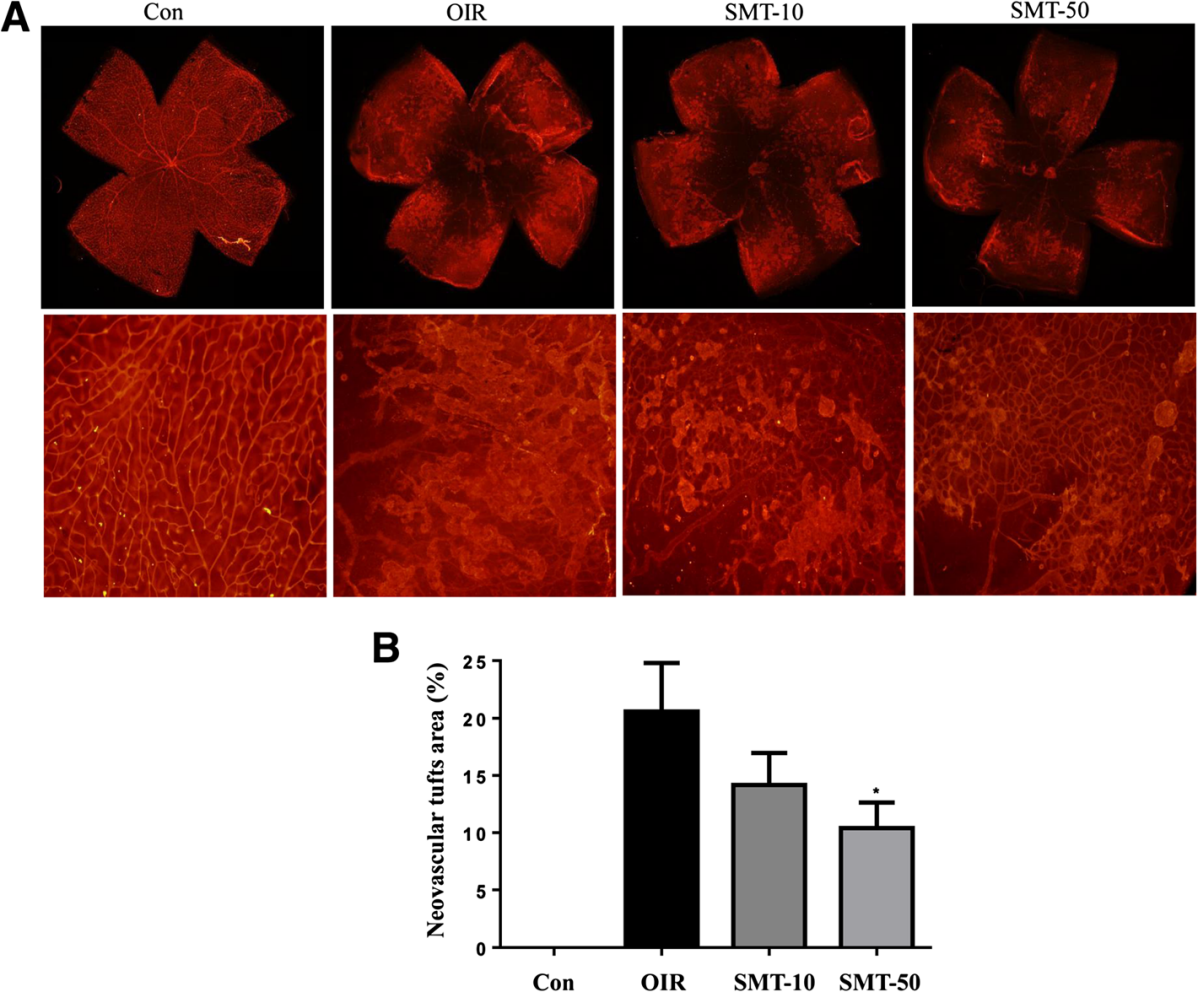
The effect of SMT on retinal neovascular tufts in OIR mice. **a** The retinal neovascular tufts were visualized using isolectin B4 staining. Con, normal control mice; OIR, saline-treated OIR mice; SMT-10, OIR mice treated with 10 mg/kg of SMT; and SMT-50, OIR mice treated with 50 mg/kg of SMT. **b** Quantification results are expressed as neovascular tufts on the retina surface. The values in the bar graph represent the mean ± SE (*n* = 5; *p < 0.05 vs. saline-treated OIR mice)

The OIR mice that were treated with SMT exhibited a significant decrease in the pathological changes that occurred during ischemic retinopathy. As shown in Figs. 1 and 2, SMT reduced the area of vascular obliteration of the central retina and prevented pathogenic retinal neovascularization compared with the OIR group on P17. Mice that were treated with 10 mg/kg of SMT failed to exhibit a significant change in the area of non-perfusion in the center of the retina, but 50 mg/kg of SMT significantly decreased the non-perfused area compared with the OIR group (Fig. 1). In addition, both doses of SMT significantly reduced the formation of neovascular tufts (by 31.1 and 49.4 %, respectively) compared with the OIR group (Fig. 2). Consequently, SMT helped prevent the pathogenic retinal neovascularization during ischemic retinopathy.

### Effect on the protein expression of angiogenesis-related factors

**Fig. 3:**
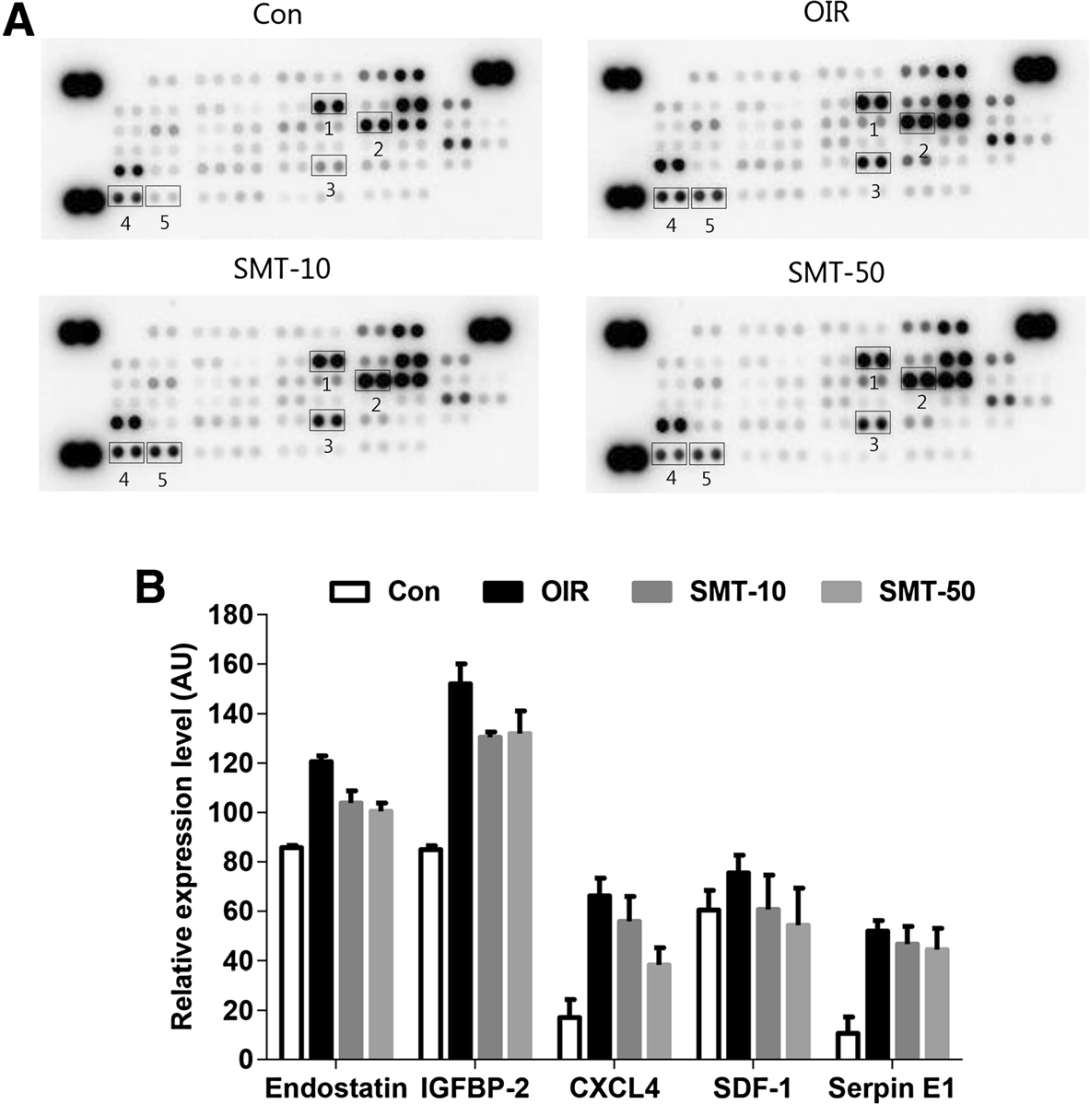
Expression of retinal angiogenesis-related proteins in OIR mice. (**a**) Proteins whose expression was modulated in retinas treated with SMT are indicated by numbers. Con, normal control mice; OIR, saline-treated OIR mice; SMT-10, OIR mice treated with 10 mg/kg of SMT; and SMT-50, OIR mice treated with 50 mg/kg SMT. 1: Endostatin; 2: IGFBP-2, insulin-like growth factor binding protein 2; 3: CXCL4; 4: SDF-1, stromal cell-derived factor 1; 5: Serpin E1. (**b**) The levels of pro- and anti-angiogenic factors in the retina were analyzed using protein arrays and quantified using ImageJ software. The positive controls are located in three corners of the arrays, and the negative control is located in the lower right corner of the arrays

SMT decreased the expression of pro-angiogenic factors (i.e., endostatin, IGFBP-2, SDF-1 and serpin E1) in a dose-dependent manner compared with saline-treated OIR mice (Fig. 3). In addition, anti-angiogenic factors, such as CXCL4, were significantly downregulated by SMT. This result demonstrates that SMT exerts an inhibitory effect on angiogenesis by downregulating the expression of endostatin, IGFBP-2, SDF-1 and serpin E1. The upregulation of CXCL4 in the OIR group may have been a defense mechanism against angiogenesis. Consistent with a previous study [20], the VEGF protein was not detected in the angiogenesis protein array. In addition, HIF-1α and CXCR4 could not be tested using this array. Thus, we further investigated changes in the gene expression level of HIF-1α, VEGF and CXCR4 at the mRNA.

### Effects on expression of HIF-1α, SDF-1, CXCR4 and VEGF mRNAs

**Fig. 4:**
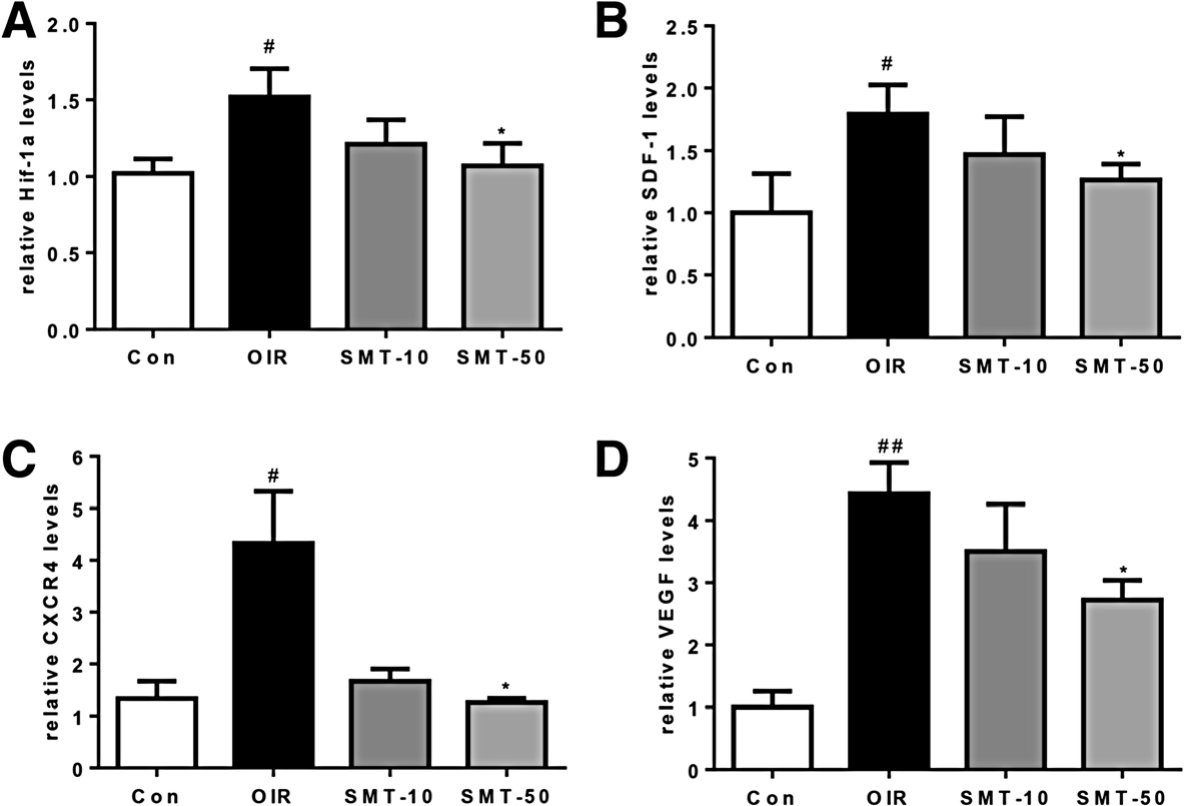
Real-time PCR analysis of HIF-1α, SDF-1, CXCR4 and VEGF mRNA levels in OIR mice. When compared with normal controls, the relative expression levels of the HIF-1α (**a**), SDF-1 (**b**), CXCR4 (**c**) and VEGF (**d**) mRNAs were markedly increased in the retinas of OIR mice and dramatically reduced after SMT treatment. Con, normal control mice; OIR, saline-treated OIR mice; SMT-10, OIR mice treated with SMT (10 mg/kg); and SMT-50, OIR mice treated with SMT (50 mg/kg). The data are shown as the mean ± SE (*n* = 4; #$p$ < 0.05, ##$p$ < 0.0001 vs. the normal control mice, *p < 0.05 vs. saline-treated OIR mice)

The retinal mRNA expression levels of HIF-1α, SDF-1, CXCR4 and VEGF were examined using real-time PCR. In OIR mice, the HIF-1α, SDF-1, CXCR4 and VEGF mRNA levels were elevated compared with those of normal control mice. However, mRNA levels of HIF-1α, SDF-1, CXCR4 and VEGF were significantly decreased in SMT-treated OIR mice (Fig. 4).

## Discussion

The OIR model is widely used to study the pathological angiogenesis that results from ischemia. In the OIR model, the exposure of pups to hyperoxia results in endothelial cell death and vaso-obliteration, which leads to physiological hypoxia [23]. When the mice are returned to room air (P12-P17), the retina becomes hypoxic due to the absence of the retinal vasculature; this hypoxia strongly stimulates pro-angiogenic factors and results in abnormal neovascularization [23, 24]. In this study, we investigated whether SMT could inhibit retinal neovascularization in OIR mice. We demonstrated that SMT has an inhibitory effect on the expression of HIF-1α, SDF-1, CXCR4 and VEGF in OIR mice. Our findings indicate that SMT has anti-angiogenic activity during pathological retinal neovascularization. In our array of angiogenesis-related factors, the OIR mice showed upregulated protein expression of endostatin, IGFBP-2, CXCL4, SDF-1 and serpin E1. The upregulation of CXCL4, an anti-angiogenic factor, in OIR may be a defense mechanism against angiogenesis, and a decrease of CXCL4 by the SMT is associated with the mechanism by which CXCL4 inhibits the binding of VEGF [25]. In proliferative diabetic retinopathy, an increase of anti-angiogenic factors, such as sVEGFR-1 and TSP-2, may be a protective mechanism against the progression of angiogenesis [26]. To elucidate the various signaling molecules and growth factors involved in the anti-angiogenic effect of SMT in the OIR mice, we focused on HIF pathway-mediated expression.

Hypoxia inducible factor (HIF)-1α is an important oxygen-dependent regulator in both the vaso-obliterative phase, in which HIF-1α is suppressed, and the neovascularization phase, in which HIF-1α produces angiogenic factors, such as vascular endothelial growth factor (VEGF) and stromal cell-derived factor 1 (SDF-1) [27, 28].

Although VEGF is widely recognized as an ideal target for regulating angiogenesis therapies [29, 30], recent evidence has suggested that SDF-1 is a key player in ischemic retinopathies, such as proliferative retinopathy and retinopathy of prematurity [31]. SDF-1 and CXCR4 are expressed in the ischemic retina and contribute to the recruitment of bone marrow-derived endothelial cells to reduce macrophage influx and retinal neovascularization [32]. The SDF-1 expression levels are upregulated in the vitreous humor in ischemic ocular diseases, such as proliferative diabetic retinopathy and retinopathy of prematurity [33, 34]. Eyes treated with the CXCR4 inhibitor N-(1-methyl-1-phenylethyl)-N-[((3S)-1-{2-[5-(4H-1,2,4-triazol-4-yl)-1H-indol-3-yl]ethyl}pyrrolidin-3-yl)methyl] amine have significantly less retinal neovascularization on the surface of the retina [35]. In addition, several studies have suggested that the SDF-1/CXCR4 axis regulates VEGF secretion and expression, thereby promoting VEGF-mediated angiogenesis. The secretion of VEGF is induced by SDF-1/ CXCR4 activation in several cell lines. The SDF-1/CXCR4 axis augments VEGF expression at both the mRNA and protein levels, and the inhibition of SDF-1/CXCR4 signaling reduces VEGF expression in an *in vivo* model [28, 36]. Furthermore, SDF-1 and VEGF interact synergistically to promote vascular endothelial cell functions, such as cell survival, cell migration and changes in gene expression [37, 38]. In the retinas of OIR rats, VEGF and SDF-1 act synergistically to induce angiogenesis. In rats, SDF-1 induces Müller cell activation, thereby stimulating the secretion of VEGF; in turn, VEGF secretion by the ischemic retina increases CXCR4 expression in Müller cells [28]. Thus, increased levels of SDF-1 have been suggested to promote the interaction with CXCR4 and activate VEGF expression in the retina of OIR mice. Therefore, HIF-1α, SDF-1, CXCR4 and VEGF have been proposed as targets for treating retinal neovascularization. Our study demonstrated that SMT prevents retinal neovascularization and reduces the expression of HIF-1α, SDF-1, CXCR4 and VEGF in OIR mice. These results indicate that the expression of HIF-1α, SDF-1, CXCR4 and VEGF is highly correlated with retinal neovascularization and that the inhibitory effect of SMT on retinal neovascularization might occur due to the downregulation of both SDF-1 and CXCR4.

SMT contains *A. gigas*, *C. officinale*, *P. lactiflora* and *R. glutinosa. A. gigas* extract and its two major coumarins, which were previously identified as decursin and decursinol angelate, have been demonstrated to exert inhibitory effects on angiogenesis by reducing VEGF expression [39, 40]. Extracts of *C. officinale* with *Tabanus bovinus* inhibited the proliferation of glomerular capillary endothelial cells and reduced neovascular formation in the chorioallantoic membrane and the rat cornea [41]. *P. lactiflora* extracts inhibited the proliferation, migration, and tube formation ability of human vascular endothelial cells and reduced vascular density and formation in a chorioallantoic membrane model system [42]. *R. glutinosa* extracts enhanced the migration, mobilization and therapeutic angiogenesis of endothelial progenitor cells after myocardial infarction by activating the SDF-1/CXCR4 cascade [43]. These observations suggest that the prevention of retinal neovascularization by SMT may occur due to a combination of the effects of these four herbs. However, the detailed mechanism that underlies the synergistic effects of SMT on retinal neovascularization remains unknown.

## Conclusion

In conclusion, we demonstrated for the first time that SMT inhibits the retinal pathogenic angiogenesis induced by ischemic retinopathy in OIR mice. In addition, the overexpression of HIF-1α, SDF-1, its receptor CXCR4, and VEGF were significantly inhibited by treatment with SMT. These observations suggest that SMT regulates a HIF-1α and acts through an anti-SDF-1/CXCR4 and anti-VEGF mechanism to prevent retinal pathogenic angiogenesis. Taken together, these findings indicate that SMT could be a useful herbal medicine for treating ischemic retinopathy.
